# Single‐shot single‐voxel lactate measurements using FOCI‐LASER and a multiple‐quantum filter

**DOI:** 10.1002/nbm.3276

**Published:** 2015-03-19

**Authors:** Geoffrey S. Payne, Nandita M. deSouza, Christina Messiou, Martin O. Leach

**Affiliations:** ^1^ Cancer Research UK Cancer Imaging CentreRoyal Marsden Hospital and Institute of Cancer ResearchSuttonSurreyUK

**Keywords:** Spectroscopic localization, MR Spectroscopy (MRS) and Spectroscopic Imaging (MRSI) Methods, lactate, multiple quantum filter, FOCI, LASER, brain

## Abstract

Measurement of tissue lactate using ^1^H MRS is often confounded by overlap with intense lipid signals at 1.3 ppm. Single‐voxel localization using PRESS is also compromised by the large chemical shift displacement between voxels for the 4.1 ppm (–CH) resonance and the 1.3 ppm –CH_3_ resonance, leading to subvoxels with signals of opposite phase and hence partial signal cancellation. To reduce the chemical shift displacement to negligible proportions, a modified semi‐LASER sequence was written (“FOCI‐LASER”, abbreviated as fLASER) using FOCI pulses to permit high RF bandwidth even with the limited RF amplitude characteristic of clinical MRI scanners. A further modification, MQF‐fLASER, includes a selective multiple‐quantum filter to detect lactate and reject lipid signals.

The sequences were implemented on a Philips 3 T Achieva TX system. In a solution of brain metabolites fLASER lactate signals were 2.7 times those of PRESS. MQF‐fLASER lactate was 47% of fLASER (the theoretical maximum is 50%) but still larger than PRESS lactate. In oil, the main 1.3 ppm lipid peak was suppressed to less than 1%. Enhanced suppression was possible using increased gradient durations. The minimum detectable lactate concentration was approximately 0.5 mM. Coherence selection gradients needed to be at the magic angle to avoid large water signals derived from intermolecular multiple‐quantum coherences. In pilot patient measurements, lactate peaks were often observed in brain tumours, but not in cervix tumours; lipids were effectively suppressed.

In summary, compared with PRESS, the fLASER sequence yields greatly superior sensitivity for direct detection of lactate (and equivalent sensitivity for other metabolites), while the single‐voxel single‐shot MQF‐fLASER sequence surpasses PRESS for lactate detection while eliminating substantial signals from lipids. This sequence will increase the potential for *in vivo* lactate measurement as a biomarker in targeted anti‐cancer treatments as well as in measurements of tissue hypoxia. Copyright © 2015 John Wiley & Sons, Ltd.

**Abbreviations used**
*FOCI*: *frequency offset corrected inversion*
*LASER*: *localization by adiabatic selective refocusing*
*fLASER*: *LASER using FOCI pulses*
*MQF*: *multiple‐quantum filter*
*PRESS*: *point resolved spectroscopy*
*SAR*: *specific absorption rate*

## Introduction

Lactate is potentially an important biomarker of tissue hypoxia and for tumour evaluation and response. While normal tissue concentrations of lactate are low, varying between about 0.1 mM in the fully fasted state and about 1.5 mM following a high‐carbohydrate meal, blood concentrations may rise to 6 mM during exercise 1. Tissue concentrations are elevated in many tumours, including brain 2, 3, prostate 4, and squamous cell carcinoma 5.

Elevated lactate concentration in tumours is predominantly a consequence of the Warburg effect, in which tumour cells undergo glycolysis even in the presence of oxygen. Sometimes lactate accumulation is enhanced by low perfusion, reducing wash‐out of the lactate produced. Preclinical and clinical studies have shown that elevated tumour lactate concentrations can also be associated with subsequent development of nodal and distant metastatic disease 6, 7, 8, 9, 10. Tumour lactate concentrations are affected directly or indirectly by several chemotherapeutic agents 11 and mechanism‐based anticancer drugs, such as phosphoinositide 3‐kinase inhibitors 12, dichloroacetate 13, and Mek inhibitors 14. Inhibitors of mono‐carboxylate transporters 15 would also be expected to affect tumour lactate concentrations.

Measuring lactate using ^1^H MRS in tumours *in vivo* is hampered by the spectral overlap with lipids (see below). This is particularly a problem outside the brain or close to the skull. Most reported studies have been in brain tumours, where lipids tend to be relatively low, motion is limited, and lactate is present at concentrations of up to 14 mM 3, 16, 17, 18, 19. Lactate tends to be higher in high‐grade tumours and in metastases 3. A study in paediatric diffuse brainstem gliomas showed that response to treatment was associated with a reduction in lactate concentration, whilst no change was detected in non‐responders 19. Lactate concentration has also been found to depend on the degree of oxygenation: healthy subjects breathing 12% O_2_ exhibited a 39% increase in brain lactate compared with breathing 21% O_2_ 20. Outside the brain, pilot measurements have detected lactate in non‐Hodgkin lymphoma 21, but in prostate tumours the concentration of lactate has been found to be below the threshold for detection 22.

Lactate is an AX_3_ spin system characterized by a –CH_3_ doublet peak at 1.33 ppm and a –CH quartet at 4.1 ppm, with a *J*‐coupling constant of 6.9 Hz. The 1.33 ppm doublet is the preferred resonance for measurement since it is larger, narrower, and not affected by water suppression pulses at 4.7 ppm. One difficulty in measurement is that lactate suffers from the chemical shift displacement artefact, which causes the slices selected by excitation and refocusing pulses during volume selection to be spatially shifted for the –CH quartet at 4.1 ppm relative to the –CH_3_ doublet at 1.3 ppm. In the point resolved spectroscopy (PRESS) sequence for example this leads to some sub‐volumes of the total voxel experiencing only one or zero 180^o^ refocusing RF pulses of the coupled spins, affecting phase evolution and leading to opposing phase in these regions and therefore overall partial signal cancellation 23. This effect can be mitigated by expanding the nominal voxel size and then using saturation slices to eliminate the unwanted signals 24 or improved broadband RF pulse design 25.

Measurement of the 1.3 ppm peak using ^1^H MRS is confounded in many cases by intense overlapping signals from lipids. Many options have been proposed to selectively detect lactate signals in these cases, most exploiting the *J*‐coupling between the –CH_3_ and the –CH resonances, although the relatively long *T*_2_ of lactate compared with lipid has also been used 26. The lactate resonance also overlaps with a smaller macromolecule resonance, which also needs to be taken into account 20.

For applications *in vivo* the criteria to be considered include good elimination of lipid signals, good retention of lactate signals, good tolerance to motion, and capability to localize to the tissue of interest.

Employing *long echo times* to favour lactate detection over lipid is simple but insufficient to eliminate the strong lipid signals often found in tumours. In *spin‐echo difference editing*, selective 180° RF pulses at the frequency of the 4.1 ppm methine spins are applied during alternate scans, which affect the phase evolution of the 1.3 ppm lactate doublet of interest; subtracting these scans causes signals from lactate to add and from singlets to cancel 27, 28, 29. However, small tissue motion leads to incomplete subtraction of the large lipid signals. This makes the method possible for measurements in the head 17, 30, but not where motion is present.

*Multiple‐quantum filters* (MQFs) separate single‐quantum coherences of non‐coupled metabolites from multiple‐quantum coherences of coupled spins (e.g. lactate) using phase‐cycling or gradient pulses. Using gradient pulses for coherence selection permits lactate editing in a single shot, but in the basic implementation loses 75% of the signal. Strategies have been suggested to increase MQF lactate signals, with up to 100% lactate signal recovery in an unlocalized single acquisition demonstrated on a high‐field preclinical system 31. However, on clinical systems the best that has been reported to date is 50% in a single acquisition, or 100% using a phase cycle 32. In general, the initial excitation pulse can be made slice selective without affecting coherence pathways, while MRSI can be achieved by including appropriate phase encoding. Originally implemented on a high‐field animal scanner, a more recent publication has extended the MRSI localization to multi‐slice using Hadamard encoding on a clinical scanner, and has demonstrated this in a non‐Hodgkin lymphoma 21. A single‐slice version has demonstrated ability to detect lactate in the presence of otherwise‐dominant lipid resonances in patients with high‐grade glioma 33.

While MRSI methods have good coverage, they are subject to errors in the presence of motion, and have relatively large minimum acquisition times owing to the number of phase encode steps involved. An option for single‐voxel localization for lactate is therefore desirable. Unfortunately, incorporating an MQF into a PRESS sequence does not work, owing to the chemical shift displacement artefact 34.

The aim of the present work therefore was to develop and evaluate a single‐shot single‐voxel measurement of tumour lactate as a potential non‐invasive imaging biomarker of disease and response. The strategy was firstly to modify a semi‐LASER (localization by adiabatic selective refocusing) sequence, by replacing the hyperbolic secant RF pulses with FOCI (frequency offset corrected inversion) RF and gradient pulses. FOCI pulses produce very sharp slice profiles with high RF bandwidth, but unlike hyperbolic secant pulses do not require a high RF amplitude 35, 36 and are therefore compatible with standard clinical MRI systems. They should largely eliminate the chemical shift displacement problem. A similar strategy has been reported for single‐voxel localization 37, MRSI 38, and MEGA editing 39 in brain, and for MRSI in prostate 40, 41. An MQF module was then inserted in the centre of this sequence to selectively detect lactate signals while suppressing signals from the 1.3 ppm resonance of lipids. The implementation of this method is described, together with evaluation in test objects and a pilot cohort of patients with brain and cervical tumours *in vivo*.

## Methods

**Figure 1:**
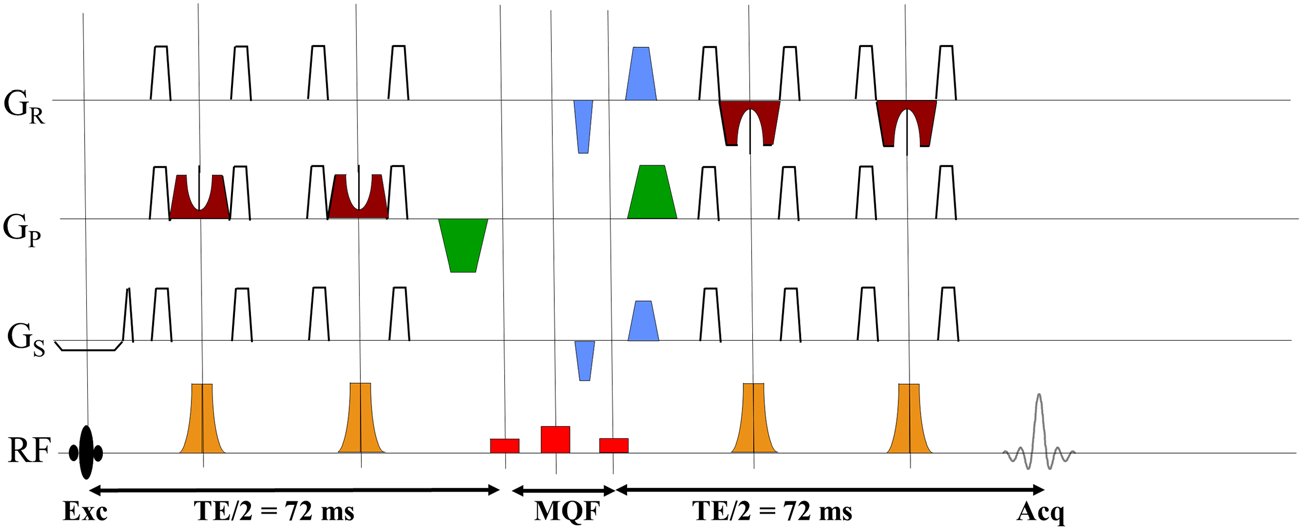
MQF‐fLASER sequence timing diagram. The initial slice‐selective excitation is followed by a pair of FOCI combined RF (brown) and gradient (maroon) refocus pulses. The MQF is positioned at *T*_E_/2, comprising rectangular 90^o^–180^o^–90^o^ RF pulses (shown in red) and coherence selection gradients in the ratio −1:2 at the magic angle (shown in blue). Additional crusher gradients (shown in green) are positioned around the MQF segment. A further pair of slice‐selective FOCI pulses is followed by acquisition.

Semi‐LASER sequences comprise slice‐selective excitation followed by two pairs of identical adiabatic inversion RF pulses (usually hyperbolic secant) with appropriate slice‐select and spoiler gradients 6. Unfortunately, the maximum pulse amplitude permitted by the hardware limits the maximum achievable spectral bandwidth; on our 3 T Philips Achieva TX system the maximum available *B*_1_ on the body coil (13.5 μT) limits the maximum hyperbolic secant pulse bandwidth to about 1200 Hz, resulting in poor slice profiles and a 1/3 voxel shift between lactate CH and CH_3_. We therefore implemented 12 kHz FOCI RF‐and‐gradient pulses to replace them (indicated in brown and maroon in Fig. 1). We will refer to semi‐LASER with FOCI pulses as “FOCI‐LASER” (abbreviated fLASER).

The MQF‐fLASER sequence (Fig. 1) was created by additionally inserting an MQF segment (based on that of Mellon *et al*. 21) at *T*_E_/2. Originally, we used similar RF pulses to Mellon *et al*. for this, using 6 ms sinc‐gauss frequency‐selective 90^o^ and 180^o^ RF pulses, with the 90^o^ pulses centred at the frequency of water and lactate –CH and the 180^o^ pulse offset by 434 Hz (lactate –CH_3_). The purpose of using frequency‐selective RF pulses for this was to affect one group of lactate spins but not the coupled partners, and hence reduce the contribution of lipid (which has no coupled spins at 4.1 ppm). We found that rectangular pulses with appropriate durations (2.714 ms for 90^o^ and 2.408 ms for 180^o^) worked at least as well in the MQF‐fLASER sequence as the sinc‐gauss pulses and also enabled us to reduce the total mixing time, which can be a significant source of signal loss 32. It was also found that for the MQF‐fLASER sequence (but not for a 2D MRSI MQF sequence such as that of Mellon *et al*.) centring all three pulses at −90Hz on the lactate –CH resonance improved signal detection. Coherence selection gradients (shown in blue) in the ratio −1:2 were placed before and after the second rectangular 90^o^ pulse of the MQF. These were at the magic angle (54.7^o^ to *z*) to avoid refocusing intermolecular multiple quantum coherences from water 42. A further pair of spoiler gradients was placed around the MQF period along *y* (green), which needed to have the same polarity when the MQF refocus pulse was centred at the frequency of the lactate –CH_3_, and opposite polarity when the MQF refocus pulse was at the frequency of the lactate –CH resonance.

Measurements were made on a 3 T Philips Achieva TX MRI system (Best, The Netherlands). Phantom measurements used the eight‐channel SENSE Head coil. The fLASER and MQF‐fLASER sequences were compared with the standard PRESS sequence (RF bandwidth 1200 Hz) in a 15 cm diameter spherical phantom of brain metabolites, including 5 mM lactate, and in a 50 ml tube of safflower oil. All phantom measurements were acquired with voxel size (2 cm)^3^, Number of signal averages (NSA) = 128, *T*_R_ = 1000 ms, *T*_E_ = 144 ms, 2048 sampling points over a 4 kHz bandwidth. All scans were acquired using “frequency stabilization”, which measures and corrects the water frequency before each acquisition to offset effects of scanner drift during the scan. This made a significant improvement to data quality. Lactate signal intensity in the phantoms was measured both using peak amplitude (after zero‐filling twice, and apodizing with a 1 Hz line broadening), and the Quest algorithm in jMRUI 43, 44, 45.

Pilot data using the fLASER and MQF‐fLASER sequences were acquired from seven patients with brain tumours (six glioblastomas, one meningioma, voxel sizes 8–15 cm^3^) and seven patients with cervical cancers (voxel sizes 3.8–15.6 cm^3^) within a protocol approved by our institutional review board. fLASER spectra without water suppression were also acquired to enable the use of tissue water as a concentration reference. Brain scans were acquired using the eight‐channel SENSE Head coil while scans on cervical cancer patients were acquired using combined external loop coils (diameter = 20 cm) and an internal coil 46 to improve signal to noise ratio. In addition one patient was scanned with a breast lesion (using the standard breast coil, and voxel size (2 cm)^3^, primarily to explore the degree of fat suppression provided by the MQF‐fLASER sequence *in vivo*), and large voxels (6 cm × 4 cm × 4 cm) were studied in the brains of two normal volunteers (to investigate whether significant signals are passed by the MQF from macromolecules at about 1.3 ppm coupled to others at about 4.3 ppm 47.

## Results

### Phantom studies

**Figure 2:**
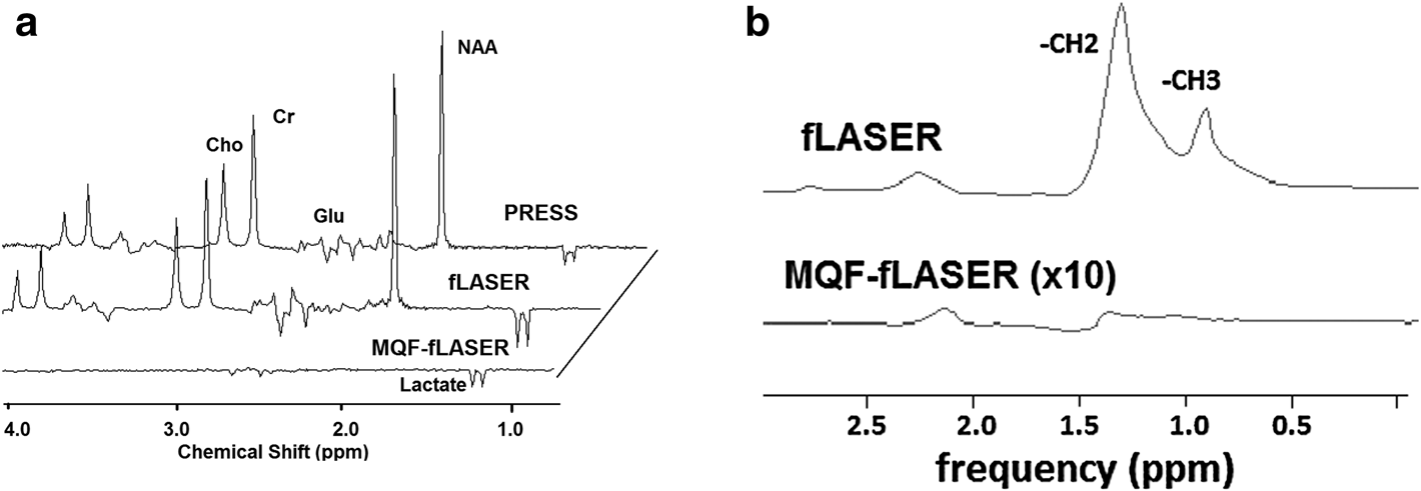
(a) PRESS, fLASER and MQF‐fLASER spectra from solution of brain metabolites (12.5 mM N‐acetyl aspartate (NAA), 10 mM creatine (Cr), 3 mM choline (Cho), 7.5 mM myo‐inositol, 12.5 mM glutamate (Glu), 5 mM lactate). (2 cm)^3^ voxel, 128 acquisitions, *T*_R_ 1000 ms, *T*_E_ 144 ms. Data were zero filled from 2048 to 4096 points, and apodized with 1 Hz line broadening. Note that while the amplitudes of the singlets (e.g. creatine) are similar in PRESS and fLASER spectra, the lactate signal is substantially larger in the fLASER spectrum as it does not suffer significant signal loss from the chemical shift displacement artefact effect. In these spectra the MQF‐fLASER lactate peak is 46% of that in the fLASER (i.e. nearly the theoretical maximum), and is also larger than that in the PRESS spectrum. (b) fLASER and MQF‐fLASER spectra of safflower oil. (2 cm)^3^ voxel, *T*_R_ = 1000 ms, *T*_E_ = 144 ms, NSA = 128 (fLASER) and 256 (MQF‐fLASER). The MQF‐fLASER spectrum is displayed after a ×10 vertical expansion. Note the almost total suppression of the lipid resonance at 1.3 ppm.

A comparison of the standard PRESS, fLASER and MQF‐fLASER spectra from the sphere of brain metabolites is shown in Fig. 2(a). The standard PRESS sequence shows all metabolites, but lactate signal is low owing to the chemical shift displacement effect, which creates subvoxels of lactate with signals of opposite phase to each other, which then cancel 26. This effect was much reduced with the fLASER sequence, with a corresponding large increase (×2.7) in detected lactate signal. The MQF‐fLASER eliminated singlets but retained significant signal from lactate. Using the lactate signal of the fLASER signal as a reference, the MQF‐fLASER sequence yielded 47 ± 1% lactate signal (based on peak area; 46 ± 1% based on peak height, *N* = 5), while the PRESS signal yielded 37 ± 5 % lactate signal (based on peak area; 38 ± 1% based on peak height, *N* = 5). Thus the MQF‐fLASER lactate signals were close to the theoretical maximum of 50% 31, 48, and were about 20% larger than the standard PRESS lactate signals. Some signal is also seen from the coupled spins in glutamate, but these do not interfere with the lactate signals. The lactate signal‐to‐noise ratio (peak signal/peak–peak noise) in the MQF‐fLASER spectrum is approximately 10:1, meaning that with this hardware and these sequence parameters (2 cm cubic voxel, 2 min acquisition) the lowest detectable lactate concentration is approximately 0.5 mM.

The MQF‐fLASER spectrum of the oil phantom (Fig. 2(b)) shows signal at 1.3 ppm of 0.86% relative to the amplitude of this peak in the fLASER spectrum, demonstrating good lipid suppression. Signal from the 2 ppm allyl peak was detected as expected 21.

### *In vivo* studies

**Figure 3:**
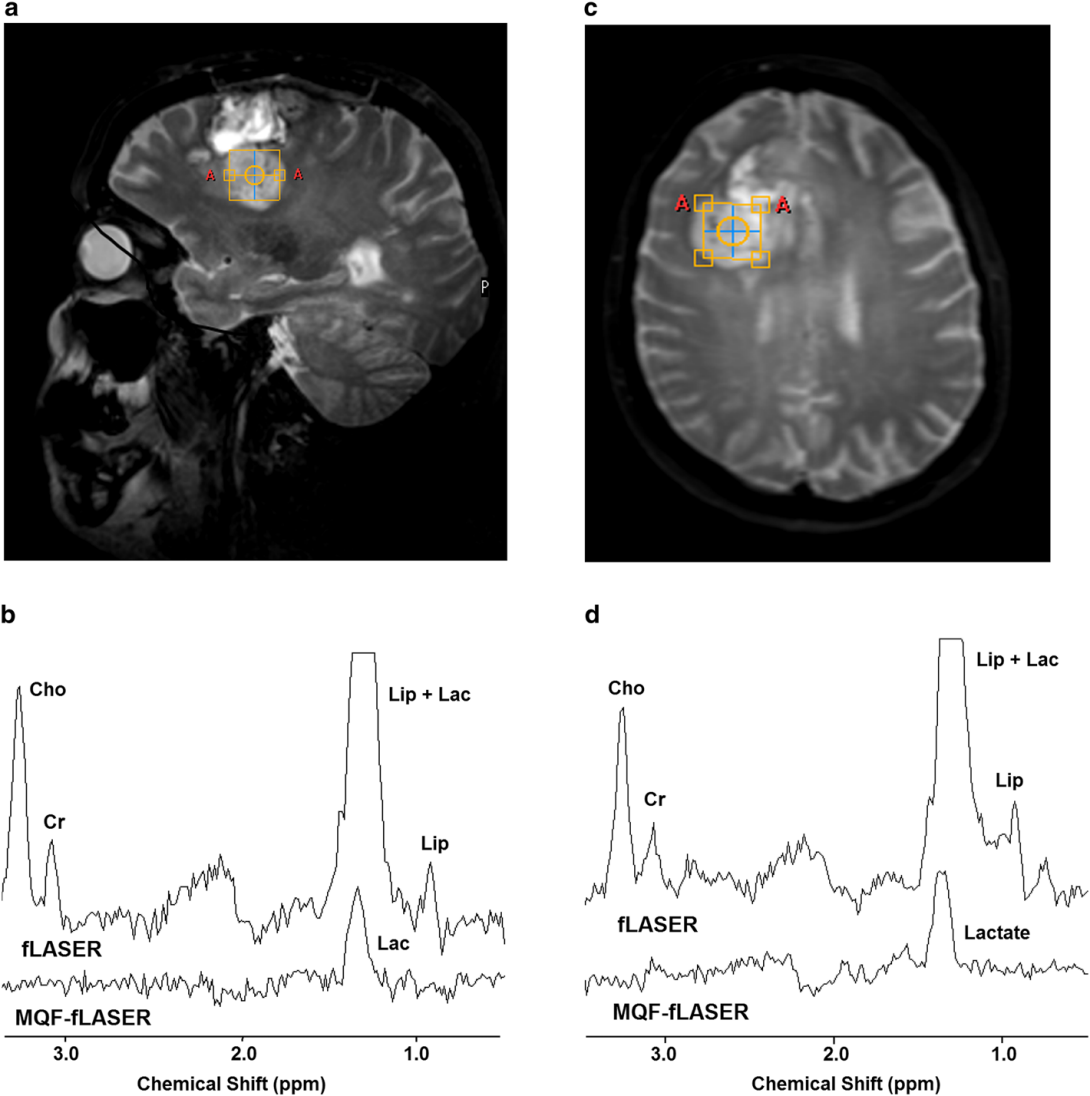
fLASER and MQF‐fLASER spectra of high‐grade brain tumours (glioblastoma multiforme). (a) Voxel location shown on sagittal *T*_1_‐weighted image (*T*_R_/*T*_E_ = 11/4.6 ms). (b) Corresponding fLASER and MQF‐fLASER spectra. Cho, choline; Cr, creatine; Lip, lipid; Lac, lactate. Water linewidth 6.0 Hz. (c) Voxel location shown on transverse *T*_2_‐weighted image (*T*_R_/*T*_E_ = 2500/229 ms). (d) Corresponding fLASER and MQF‐fLASER spectra, water linewidth 6.7 Hz. All spectra were acquired using *T*_R_ 1000 ms, *T*_E_ 144 ms, NSA 128 (fLASER) and 256 (MQF‐fLASER), and voxel size (2 cm)^3^.

Example fLASER and MQF‐fLASER spectra from brain tumours are shown in Fig. 3. In both cases the fLASER spectra show large peaks at 1.3 ppm, which must be primarily from lipid since they are in phase with the other resonances at this echo time of 144 ms. Lactate cannot be distinguished in these spectra. The MQF‐fLASER spectra also show peaks at 1.3 ppm. These can be attributed to lactate, since lipid suppression to 0.86% reduces the lipid signals to well below the noise level. In several examples of brain tumours a peak was observed at 1.3 ppm in the fLASER spectra but not in the MQF‐fLASER spectra, suggesting that these tumours contained lipid but not lactate. The concentrations of lactate were estimated assuming *T*_1_ and *T*_2_ of water were 1330 ms and 80 ms respectively 49, *T*_1_ and *T*_2_ of lactate were 1730 ms 50and 453 ms 51 respectively, brain water concentration was 41 M (following Howe et al. 3) and lactate selection efficiency was 47% (based on the phantom measurement). This resulted in approximate lactate concentrations of 7.0 mM and 9.9 mM for the brain tumours shown in Fig. 3.

**Figure 4:**
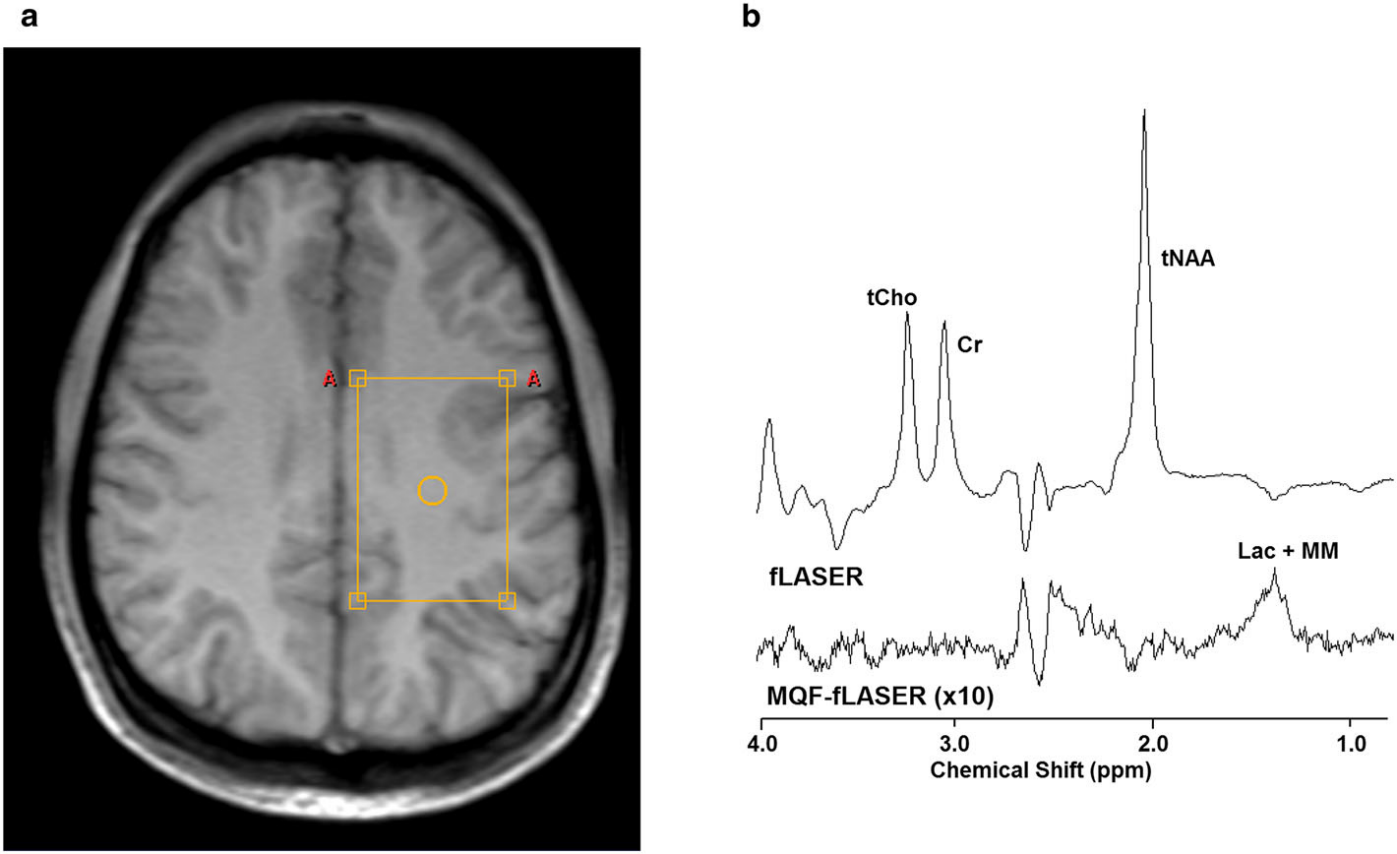
Example fLASER spectra from normal brain. (a) Voxel location shown on an axial *T*_1_‐weighted turbo field echo (TFE) image. (b) fLASER and MQF‐fLASER spectra. tCho, total choline; Cr, creatine; tNAA, total N‐acetyl aspartate; Lac, lactate MM, macromolecules. Voxel size 6 cm × 4 cm × 4 cm, *T*_R_ 2000 ms, *T*_E_ 144 ms, 4096 sample points over 4 kHz, NSA = 128 (fLASER) and 256 (MQF‐fLASER). The water linewidth was 6.4 Hz.

The MQF‐fLASER spectrum of the large normal brain voxel (Fig. 4) reveals a broad peak centred at about 1.4 ppm with a width of about 20 Hz, and a sharper doublet to the right (centred at 1.35 ppm; splitting 7 Hz). The water linewidth in this voxel was 6.4 Hz. Given the repetition time used (2 s) it is more likely that the broad component arises from macromolecules rather than from lipids. The signal‐to‐noise ratio (peak height relative to rms noise) is about 12. Thus for the usual voxel size of approximately (2 cm)^3^ the signal‐to‐noise ratio would be about unity, so not generally detectable. The corresponding peak in the spectrum without the MQF is approximately three times larger, showing that the selection efficiency of the MQF for this resonance is about 33%, which is nearly as high as for lactate. There are other resonances visible in this MQF‐fLASER spectrum of normal brain, in particular at about 2.5 ppm and 3.6 ppm. Since they are also seen in MQF‐fLASER spectra from normal brain of other volunteers, they presumably arise from other metabolites or macromolecules not totally eliminated by the MQF. While these resonances have not been assigned, they are well resolved from the peaks at 1.3 ppm and therefore should not interfere with the measurements of lactate.

**Figure 5:**
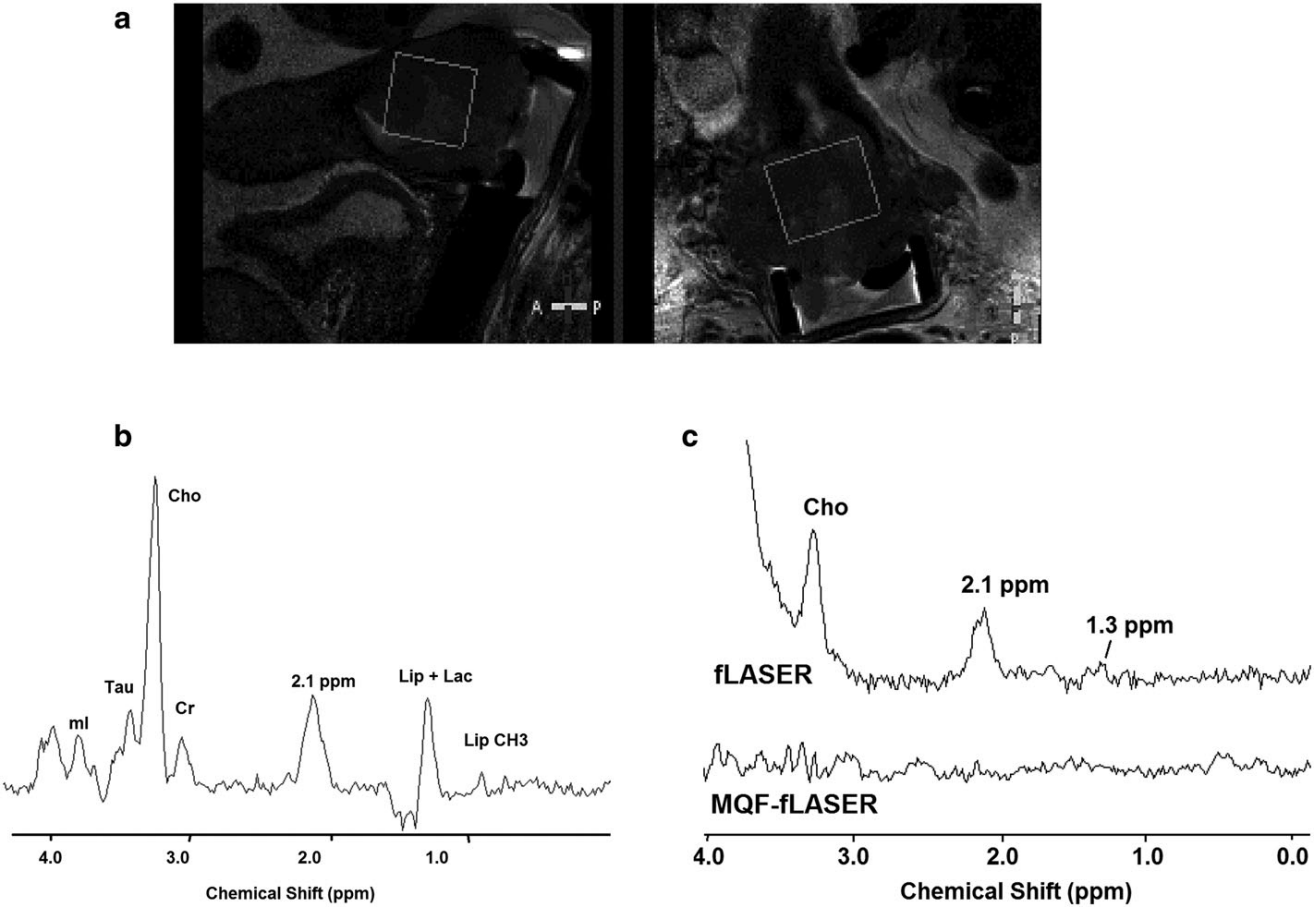
Example fLASER spectra from cervical tumours. (a) Voxel location shown on sagittal and coronal *T*_2_‐weighted images of Stage 2b cervical squamous cell carcinoma (*T*_R_/*T*_E_ = 2750/80 ms). (b) Corresponding fLASER spectrum. mI, myo‐inositol; Tau, taurine; Cho, choline; Cr, creatine; Lip, lipid; Lac, lactate. Voxel size 10 cm^3^, *T*_R_ 1320 ms, *T*_E_ 144 ms, NSA 128, water linewidth 10 Hz. (c) Example fLASER and MQF‐fLASER spectra from Stage 2b poorly differentiated squamous cell carcinoma. Note the very low levels of both lipid and lactate at 1.3 ppm in the fLASER spectrum, and the absence of any significant signals (including lactate) in the MQF‐fLASER spectrum. Voxel 3.8 cm^3^, *T*_R_ = 1346 ms, *T*_E_ = 144 ms, NSA = 128 (fLASER), 256 (MQF‐fLASER), water line width 18 Hz.

The fLASER spectra in cervix tumours (Fig. 5) are dominated by choline. There is a significant peak at 2.1 ppm that has been previously reported (source not yet identified 52, 53). The first example also shows well defined peaks from myo‐inositol, taurine, and creatine. In spite of the presence of a peak at 1.3 ppm in the fLASER spectra of some cervix tumours, no MQF‐fLASER spectra showed significant peaks at 1.3 ppm and hence did not detect any significant signals from lactate (Fig. 5(c)).

**Figure 6:**
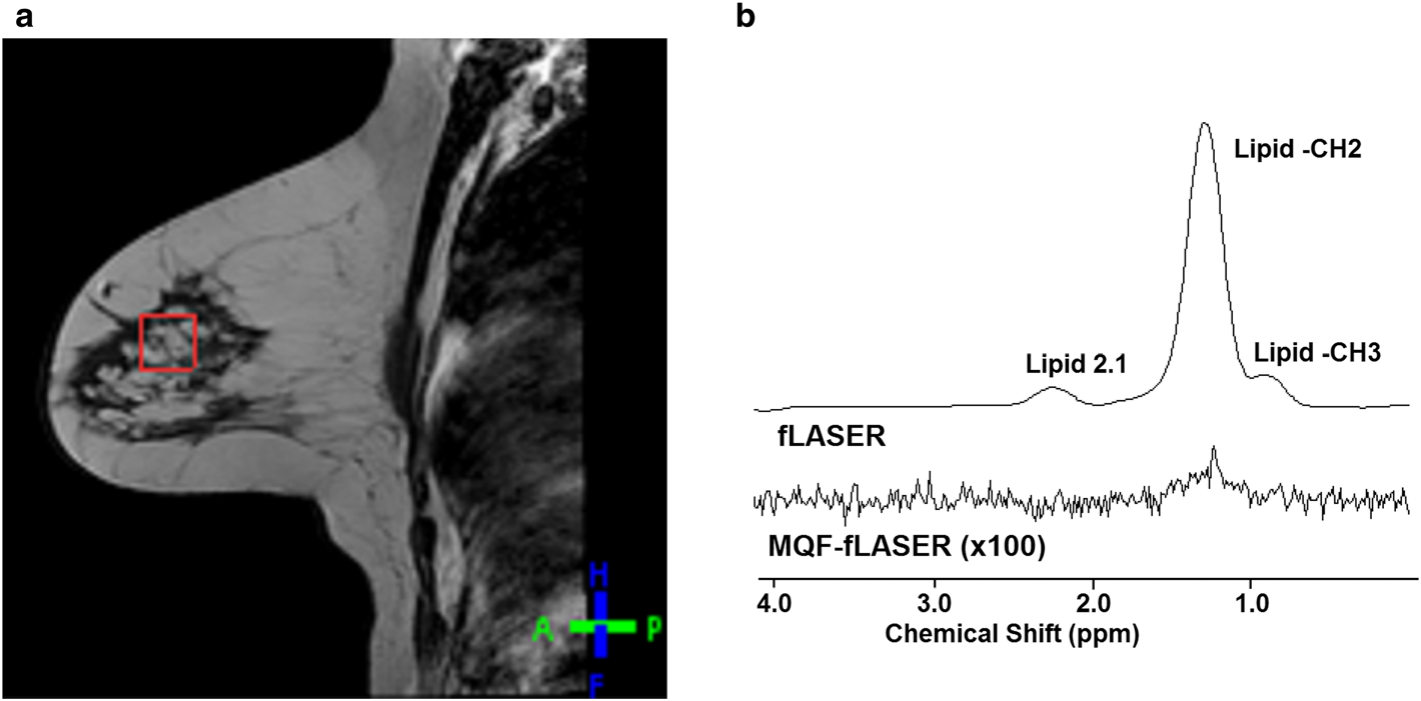
Example fLASER spectra from invasive lobular carcinoma. (a) Voxel position on sagittal *T*_1_‐weighted turbo spin echo (TSE) image (*T*_R_/*T*_E_ = 399/10 ms). (b) Corresponding fLASER and MQF‐fLASER spectra. Voxel size (2 cm)^3^, *T*_R_ = 1000 ms, *T*_E_ = 144 ms, NSA = 128 (fLASER) and 256 (MQF‐fLASER). Note the almost total elimination of the lipid peak at 1.3 ppm.

The fLASER spectrum from the breast lesion (Fig. 6(a)) is totally dominated by lipid. The peak at 1.3 ppm in the corresponding MQF‐fLASER spectrum has an amplitude of 0.16% relative to that in Fig. 6(a). It is not possible to determine what proportions of this peak are due to lactate and lipid, but it demonstrates that lipid is largely eliminated by the filter.

## Discussion

The use of FOCI pulses permitted the implementation of a semi‐LASER single‐voxel MRS localization sequence compatible with a standard clinical MRI system transmitting with a body coil with limited *B*_1_ amplitude. For the standard PRESS sequence, with RF bandwidths for the refocusing pulses of 1200 Hz, the chemical shift displacement between the lactate –CH and the lactate –CH_3_ voxels is approximately 30% (in each direction), but by using the FOCI pulses with bandwidth of 12 kHz this was reduced to approximately 3%. The relative displacement of the acquired voxels from all metabolites at different chemical shifts was therefore also much reduced, while signals from the lactate multiplet were greatly increased owing to the large reduction in the chemical shift displacement effect (Fig. 2). Thus, even without the use of an MQF, the fLASER sequence is much to be preferred compared with standard PRESS sequences for the detection of lactate. The main limitation is the increased minimum values for *T*_E_ and *T*_R_ (76 ms and 1000 ms respectively in the current implementation) owing to the number and duration of the refocusing FOCI pulses. However, for detection of lactate these are not a restriction, since for a refocused lactate doublet one requires a *T*_E_ of 144 ms, and with a *T*_1_ of about 1.73 s 50 the optimum signal‐to‐noise ratio is achieved for a repetition time of about 2.2 s.

The measurements in safflower oil demonstrated that in the current implementation the 1.3 ppm lipid peak is reduced to less than 1%. Further fat suppression is possible by incorporating longer spoiler gradient pulses, but at the expense of increasing the minimum echo time. The data from the breast tumour show that lipid is also effectively reduced under the conditions experienced *in vivo*. This shows that, if the peak at 1.3 ppm in the MQF‐fLASER spectrum has a signal greater than 1% of the lipid in the corresponding non‐filtered spectrum, it may be reasonably ascribed as predominantly arising from lactate.

Although incorporation of the MQF module into the fLASER sequence suppressed signals from singlets and retained lactate signals, the penalty for this single‐shot approach was a reduction in lactate signal intensity by a little over 50%, which is admittedly a serious loss, especially when lactate concentrations are low. However, this still exceeded the signal from the standard PRESS sequence. Methods exist to recover the full signal of lactate, using either spin echo difference methods 29 or MQFs 32, but at the expense of introducing a phase cycle. In the brain this would probably be acceptable and lead to improved signal intensity, but for extra‐cranial tumours, where there may be both increased lipid and motion, add–subtract schemes are best avoided.

A further limitation of the MQF scheme is that when no lactate signal is seen (for example in the cervical tumours) there is uncertainty as to whether this indicates that lactate concentrations are below the detection threshold, or whether one has had a technical failure. Acquisition of a few transients of the corresponding spectrum without the MQF or water suppression provides significant reassurance regarding technical aspects including shim quality and provides a water reference to aid quantification. Further acquisition of a water‐suppressed spectrum will also provide complementary information regarding other MR‐visible metabolites, but at the expense of extended measurement duration.

For the MQF‐fLASER sequence specific absorption rate (SAR) was dominated by the FOCI pulses with only 1% added by the MQF pulses, yielding a minimum *T* of about 1 s (for a maximum SAR to the head of 3.2 W/kg). It was found that placing the MQF selection gradients at the magic angle (as previously recommended 42) was definitely important to eliminate large near‐resonant artefacts otherwise observed.

As with previous reports, lactate signals were detected in some but not all of the brain tumours studied. Although *in vivo* studies measuring lactate by direct detection in brain tumours have been generally confused by overlap with lipid peaks, they do suggest a large variation in lactate concentration, for example 11.7 ± 7.0 mM in glioblastomas 3. Measurements on tissue samples do not suffer the same problem of lipid overlap, although they are compromised by the rapid changes in lactate post‐excision 54. However, these studies also suggest that there is a high degree of variation in lactate content between patients 55. The measurements in normal brain showed that, while the MQF is almost as good at selecting signals from the macromolecule peak near 1.3 ppm as it is in selecting lactate, for a standard voxel size of about (2 cm)^3^ this is unlikely to be detectable unless the signal from macromolecules is significantly elevated. When a good shim is achievable, any signals from macromolecules can often be discriminated from those of lactate on the basis of linewidth, and also on the basis of chemical shift 20; however, in some cases the contribution of macromolecules to the MQF‐fLASER peak at 1.3 ppm cannot be totally excluded.

Despite good quality spectra in the majority of the cervix tumours studied (e.g. Fig. 5(b)), no lactate MQF signals were seen. In many cases this was consistent with the absence of a lactate + lipid peak at 1.3 ppm in the fLASER direct detection spectra, showing that these tumours had no significant content of either lactate or lipid. In the other cases the lipid content was eliminated by the MQF‐fLASER sequence. The absence of significant amounts of lactate in cervical tumours was surprising, as these tumours are classically hypoxic. Although lactate signals have been reported in several *ex vivo* measurements of cervical tumour samples 56, 57, these may reflect a different population of tumours, with a contribution from post‐excision metabolism. A similar absence of significant lactate has recently been reported in prostate tumours, in which measurements at 3 T by Kobus *et al*. concluded that any lactate present must be below their detection threshold of about 1.5 mM 22.

## Conclusion

fLASER and MQF‐fLASER sequences have been implemented and demonstrated *in vitro* and *in vivo*. They are both effective at detecting lactate, while the MQF‐fLASER sequence has the advantage of suppressing co‐resonant signals from tissue lipids to less than 1%. Measurements in a pilot cohort of patients revealed lactate signals in some brain tumours but no significant lactate in any of the cervix tumours studied. Lipid was well suppressed in all cases. Use of these sequences should increase the potential of lactate measurements as a biomarker in targeted anti‐cancer treatments as well as in measurements of tissue hypoxia.
